# Cdc6 as a novel target in cancer: Oncogenic potential, senescence and subcellular localisation

**DOI:** 10.1002/ijc.32900

**Published:** 2020-02-17

**Authors:** Nicholas Lim, Paul A. Townsend

**Affiliations:** ^1^ Division of Cancer Sciences, Faculty of Biology, Medicine and Health, Manchester Academic Health Science Centre, Manchester Cancer Research Centre, NIHR Manchester Biomedical Research Centre University of Manchester Manchester United Kingdom; ^2^ Molecular Carcinogenesis Group, Department of Histology and Embryology School of Medicine, National and Kapodistrian University of Athens Athens Greece

**Keywords:** Cdc6, pancreatic cancer, senescence, subcellular localisation, cytoplasmic Cdc6

## Abstract

Cdc6 is a key replication licencing factor with a pivotal role in regulating the process of DNA replication, rendering it an important investigatory focus in tumourigenesis. Indeed, Cdc6 overexpression has been found to be a feature in certain tumours and has been associated as an early event in malignancies. With a focus on pancreatic cancer, there are evidence of its convergence in downstream pathways implicated in major genetic alterations found in pancreatic cancer, primarily KRAS. There is also data of its direct influence on protumourigenic processes as a transcriptional regulator, repressing the key tumour suppressor loci *CDH1* (E‐Cadherin) and influencing epithelial to mesenchymal transition (EMT). Moreover, gene amplification of Cdc6 as well as of E2F (an upstream regulator of Cdc6) have also been found to be a key feature in tumours overexpressing Cdc6, further highlighting this event as a potential driver of tumourigenesis. In this review, we summarise the evidence for the role of Cdc6 overexpression in cancer, specifically that of pancreatic cancer. More importantly, we recapitulate the role of Cdc6 as part of the DNA damage response and on senescence—an important antitumour barrier—in the context of pancreatic cancer. Finally, recent emerging observations suggest that the potential of the subcellular localisation of Cdc6 in inducing senescence. In this regard, we speculate and hypothesise potentially exploitable mechanisms in the context of inducing senescence *via* a novel pathway involving cytoplasmic retention of Cdc6 and Cyclin E.

**Abbreviations**
DDR: DNA damage response
DSB: double‐strand break
EMT: epithelial to mesenchymal transition
IPMN: intraductal papillary mucinous neoplasm
MEF: mouse embryonic fibroblast
OIS: oncogene‐induced senescence
PCR: polymerase chain reaction
PDAC: pancreatic ductal adenocarcinoma
SASP: senescence‐associated secretory phenotype

## Cdc6 with a Focus in Pancreatic Cancer—An Introduction

Pancreatic cancer is a relatively deadly malignancy with a 5‐year survival rate of 4% for all stages—the lowest among major solid malignancies.^1^ Surgery remains the mainstay of treatment with curative potential but only approximately 20% of presentations are surgically resectable.^2^ Despite progress made in terms of other malignancies, pancreatic cancer survival rates have remained virtually the same over the past decades.^2^ This is sobering and highlights the need for efficient and effective detection and therapeutic methods. Cancer research has been an area of intense scrutiny; however, the complex nature of this disease is a major stumbling block. Nevertheless, the core principles remain the same—progression of pancreatic cancers lie along a spectrum from precursor lesions to malignancies, which represent windows of opportunities. Critically, disease progression in cancer is linked to alterations and dysregulation of protumourigenic and tumour suppressor genes and their signalling pathways.^3^ These have clear implications in identifying important factors for timely therapeutic intervention and patient survival.

KRAS, CDKN2A, TP53 and SMAD4 are some of the genes found to have been well described and commonly mutated in pancreatic cancer, with major downstream repercussions in their signalling pathways.^3^, ^4^ Given the regulatory functions of some of these common mutations in terms of maintaining controlled cellular proliferation, it is likely that important molecules involved in the replication machinery such as Cdc6 are implicated as well. Indeed, the oncogenic potential of Cdc6 is well‐described—as part of the replication machinery, impacting genomic integrity and on the devastating outcomes of its dysregulated expression.^5^, ^6^

In addition, there are indications of Cdc6‐dependency and role in the downstream signalling pathway of KRAS‐mutant cells which are common in pancreatic cancers.^7^ With regards to KRAS, it has recently been found that the transcriptional co‐activator YAP1/Tead2 complex was upregulated in KRAS‐dependent pancreatic adenocarcinoma (PDAC) cells which relapsed after KRAS suppression.^8^ The Yap1/Tead2 complex was also shown to be an important signalling component for KRAS in epithelial to mesenchymal transition (EMT) in colon cancer cells.^9^ These indicate that Yap1/Tead 2 are potential surrogates in KRAS mutants contributing to acquired resistance to suppression by KRAS.^8^, ^9^ The significance of this is that the Yap1 complex converges on the E2F pathway, of which Cdc6 is a downstream regulatory effector that controls the cellular replication cycle.^8^ A study by Salabat and colleagues also highlighted the potential of Cdc6 as a therapeutic target, demonstrating that apigenin, a flavonoid, induced cellular arrest that was associated with a decrease in levels of Geminin (frequently upregulated in cancers and part of a regulatory mechanism of DNA replication) and more importantly that of Cdc6 in pancreatic cancer cell lines.^10^

Cdc6 is also known to act as a molecular switch with a transcriptional effect on E‐Cadherin and subsequently affecting EMT.^11^, ^12^ The aberrant expression of Cdc6 has been described to compromise the replication machinery.^13^, ^14^, ^15^ Nevertheless, what is more interesting is not merely that of its oncogenic potential *per se*, but rather also its association with senescence. Senescence is understood to be the process by which cells cease participation in the cell cycle, thereby entering a cell arrest condition.^16^, ^17^ It has been described as an effective barrier against tumour proliferation due to the aforementioned characteristics. The role of senescence as a tumourigenic barrier was further augmented with the outlining of oncogene‐induced senescence (OIS).^13^, ^18^, ^19^, ^20^, ^21^ The phenomenon of senescence needs to be overcome in order for tumours to progress, as postulated by the oncogene‐induced DNA damage model for cancer development.^22^ It is crucial that the relationship between Cdc6 and senescence is interrogated, elucidating the role of Cdc6 not only in its capacity as an oncogene but also as a molecule to be exploited in inducing senescence.

Given the potential of Cdc6 participation in pancreatic cancer, this review seeks to recapitulate and provide a brief overview of Cdc6 and its oncogenic potential, emphasising particularly its participation as part of a wider senescence network. More crucially, this review seeks to further interrogate the prospects of the interplay between the subcellular localisation of Cdc6 and senescence, and in the process, hypothesising a novel pathway for Cdc6‐induced senescence.

## Cdc6 as an Oncogene

The process of cellular replication is one that is fraught with danger and precisely regulated—the accurate replication of DNA within a cell is critical for its own survival and to ensure the passage of genetic material to progeny in a filial manner.^6^, ^23^ This process is regulated in part under tight supervision of replication licencing factors—of which Cdc6 is a key molecule.^5^, ^6^, ^23^ Indeed, dysregulation of the replication process is associated with various types of diseases due to the ensuing genetic instability.^6^ Considering its integral role in DNA replication, it is important to consider the implications of Cdc6 dysregulation in cancer. The resulting replicative stress that occurs as a result of the constraints of dysregulation potentially underlies the ensuing DNA double‐strand break (DSB),^23^, ^24^, ^25^ as proposed within the oncogene‐induced DNA damage framework.^13^, ^18^, ^22^ Indeed, some studies have indicated that neoplastic tumour growths exhibit characteristic overexpression of replication licencing factor Cdc6.^15^, ^26^, ^27^, ^28^ It has been reported that replication licencing factor Cdc6, in addition to Cdt1, was found to be overexpressed in lung carcinoma samples.^26^ In addition, observations implicating Cdc6 as a factor in contributing to increasing aggressiveness have also been reported in cervical cancer,^28^ and bladder cancer.^27^ Furthermore, high levels of Cdc6 detected immunohistochemically was not merely an indication of an increased proliferation rate due to the tumour process itself owing to its role as a replication licencing factor, but rather a product of its dysregulated expression leading to the increased proliferation—there was no correlation between Cdc6 and levels of Ki‐67, a proliferation marker.^15^ This observation can be further bolstered by the detection of significantly high levels of Cdc6 in early malignancies, even at the dysplastic stages—further suggesting an early participation of Cdc6 overexpression in driving malignancies.^15^ Moreover, gene amplification of Cdc6 was also confirmed in a significant proportion of tumour samples examined.^15^ While this represents an independent event for Cdc6 gene amplification, Cdc6 amplification could also potentially be the secondary result of an associated event—Cdc6 was found to be amplified in association with ERRB2(HER2) amplification.^15^ The ERRB2(HER2) gene is frequently implicated in cancers and is located in close proximity to the Cdc6 locus.^15^ Regardless, gene amplification is frequently acknowledged as a feature for oncogene activation.^15^ Furthermore, the regulation of Cdc6 is transcriptionally controlled *via* the E2F/Rb cascade.^29^, ^30^, ^31^ To this end, the upregulation of E2F proteins—which is frequently implicated in cancers^29^—could represent another source of dysregulated Cdc6 expression.^15^, ^29^ Indeed, the disruption of Rb has been demonstrated to confer a faster neoplastic transformation rate in KRAS activated cells, and with a worse prognosis. The position of Cdc6 in the Rb cascade highlights the effects of its transcriptional regulation, and subsequently its impact downstream of the cascade, implicating its role in tumourigenesis.^32^

More importantly, overexpressed Cdc6 also appears to have a transcriptional effect in terms of EMT^11^—a key hallmark of more rigorous invasiveness and metastasis in cancer^33^—in this case by repressing E‐cadherin expression.^11^, ^12^ The mechanistic basis of this appears to involve the binding of overexpressed Cdc6 to an E‐box motif located within the promoter region and displacing chromosomal insulator CTCF and turning off E‐Cadherin.^11^, ^12^ This appears to activate adjacent origins of replication, while at the same time resulting in transcriptional repression, acting as a “molecular switch”.^11^, ^12^

These are indeed significant reports as Cdc6 is now revealed to be a common conduit linking transcriptional repression and origin activation,^12^ with implications of having a more direct influence on protumourigenic properties.

## Cdc6 and Senescence

**Figure 1:**
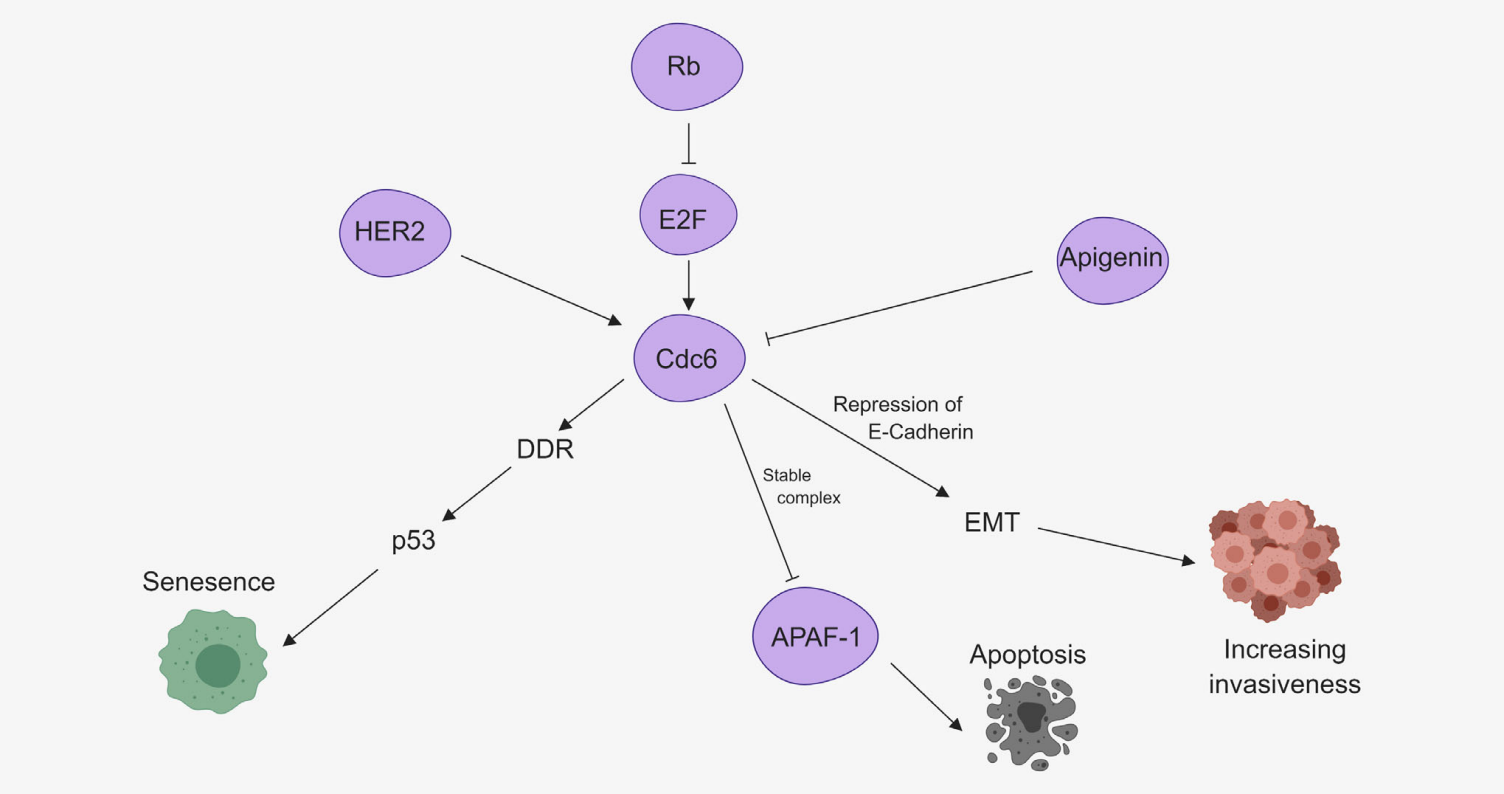
Brief overview of the oncogenic potential of Cdc6. Cdc6 control lies within the E2F paradigm^29^, ^30^, ^31^—gene amplification of E2F is a common occurrence in cancer.^29^ Cdc6 gene amplification has also been associated with HER2 amplification.^15^ Cdc6 overexpression has also been found to inhibit apoptotic pathways^38^ and represses E‐Cadherin,^11^ promoting EMT leading to increasing invasiveness.^33^ Cdc6 has also been found to induce senescence when overexpressed^13^, ^14^—Created with BioRender.com. [Color figure can be viewed at wileyonlinelibrary.com]

The oncogene‐induced DNA damage model of cancer development helps explain key features of tumour promotion; the underlying basis for the genomic instability that characterises the chromosomal instability in cancers, and the DDR in premalignant lesions that constitute a barrier to cancer progression.^13^, ^19^, ^20^, ^22^ Primarily, the activation of hyper‐proliferative signals overwhelm the replication machinery leading to replicative stress, resulting in DNA damage in the form of DNA DSB.^22^ Overexpression of oncogenes have been found to induce DNA DSBs in these cells from the earliest stages of malignancies.^15^, ^26^ Furthermore, the DDR has been found to induce a barrier to cancer progression *via* multiple checkpoint pathways—most likely *via* the p53 dependent ones–and progression is incumbent upon the bypass of these barriers.^13^, ^22^, ^34^ Essentially, an oncogene‐induced DNA damage occurs—triggering replicative stress in the form of DNA DSB—leading to a DDR, the bypass of which is critical in tumour progression.^13^, ^19^, ^20^, ^22^ There are a few key points to be made about senescence and its association in cancers; senescence markers have been found in precancerous lesions but not in full blown malignancies—suggesting that it is potentially an effective barrier induced by DNA damage to halt cellular proliferation, and in cancerous cells, the effectors and mediators of such a DDR have been overcome or selectively repressed.^13^, ^21^, ^22^, ^34^ More specific to pancreatic cancer, markers of senescence have been reported to be detected in intraductal papillary mucinous neoplasms (IPMN—precursor lesions) of the pancreas.^35^ In concordance with previous reports, there is a demonstrable attenuation of the senescence response as the lesion progresses to invasive PDAC.^35^ Furthermore, p16^INK4^ was also noted to be expressed in IPMN but gradually attenuated as senescence faded.^35^ This may be hardly surprising, given that p16/CDKN2A is noted to be a commonly mutated feature in Pancreatic Cancer.^3^ Of note, Cdc6 on one hand can suppress the p16^INK4A^ locus,^11^ while at the same time is found aberrantly overexpressed from the earliest stages in epithelial carcinogenesis,^12^ providing an alternative explanation of senescence bypass in pancreatic cancer. Interestingly however, is the fact that studies have noted that senescence triggered by oncogenic RAS is associated with accumulation of p16 and p53—explaining the selective mutations of these genes in cancers.^36^ Senescence is fast becoming a trending target in cancer studies due to its potency and with this, an interesting question arises: what is it that allows the premalignant cells to escape senescence and progress to cancers? With regards to Cdc6 and senescence, we can see now the associations and links between the two. Interestingly, Cdc6 has been shown to induce senescence and arrest proliferation in cellular models.^14^ An epithelial cellular model generated in this case was believed to be highly representative of tumours as most are epithelial in origin.^14^ This model by Komseli *et al*. utilised GL13, commercially available as SenTraGor™, which readily detects lipofuscin, as a biomarker for senescence, far increasing the versatility of the model.^14^ This is particularly important to note as although SA‐β‐Galactosidase have been widely alluded to in various studies as a marker of OIS, there are practical limitations to its use.^14^ The versatility of the model developed by Komseli *et al*. enables a far more robust utility, which enables not only a detection of senescence but also as a tracking marker of cells which have escaped senescence—a key feature in understanding tumourigenesis, as demonstrated in the prototypical tool which exhibits the interplay between cell, tissue, senescence and transcription regulation, in which Cdc6 appears to be the common theme.^14^, ^37^ Furthermore, while apoptosis is also a highly effective barrier and attractive target in cancer chemotherapy, recent reports specific to Cdc6 have indicated that Cdc6 inhibits the assembly of apoptosomes—the pilot of apoptosis—possibly by forming stable Apaf‐1 complexes.^38^ This highlights the significance of senescence as a target in Cdc6‐overexpressing tumours—senescence appeared to be the only mechanism arising to prevent Cdc6‐induced proliferation in the study by Komseli *et al*.^14^ This is also intriguing considering how most pancreatic cancers appear to be resistant to conventional apoptosis‐inducing chemotherapy regiments.^39^ Furthermore, a key feature arising in the study was that a subset of cells eventually began proliferating and demonstrated undetectable levels of markers of senescence, termed the escaped cells.^14^ These findings appear to reinforce what we know so far; (*i*) that senescence is a key feature of premalignant cells, functioning as a barrier to proliferation upon some form of oncogenic stress,^13^, ^18^, ^20^, ^34^ and (*ii*) progress to malignancy is dependent on the bypass of senescence.^13^, ^14^, ^20^, ^34^ Taking all this into consideration, we can begin to see a bigger picture with regards to the oncogenic potential of Cdc6 (Fig. 1).

It is also important to note that senescence is not a static feature as an “end‐all”, as there is growing evidence that senescence is part of a wider tissue ecosystem and not merely a confined cellular activity.^17^, ^18^, ^40^, ^41^ While senescence inhibits cellular proliferation, it has been implicated in both tumour suppressor and inhibitory circumstances,^17^, ^18^ in which lies the complexity of the senescence associated secretory phenotype (SASP) which varies with cellular context.^41^ As has been alluded to, senescence and tumourigenesis is part of a wider stromal activity that is not boxed‐in within any individual cell.^14^, ^17^, ^18^, ^41^ Indeed, central to this theme is the SASP, exhibited by cells undergoing senescence, where the focus appears to not be on a cellular level but rather in affecting the tumour microenvironment in an autocrine and paracrine manner.^16^, ^17^, ^18^ The implications of the SASP are wide‐ranging and seemingly counter‐intuitive—from tumour‐suppression to ageing, healing and tumourigenesis.^17^ Potentially, the question remains if senescence is indeed a double‐edged sword in the fight against cancer. Nevertheless, it is apparent that chronic accumulation of senescent cells are detrimental and the capacity of senescence and SASP as part of a true antitumour secret weapon relies on being given the ammunition of immunoclearance.^37^, ^40^, ^42^ Furthermore, the potential for senescent cells (long thought to be a permanent state) to escape highlights the implications of the SASP on the cellular microenvironment and the features leading to tumourigenesis.^14^ Nevertheless, the first step toward building an arsenal is to induce the vulnerability to immune‐clearance and halting cell proliferation^40^—senescence—and how we can exploit it. Indeed as alluded to by Myrianthopoulos and colleagues, senotherapeutics is an exciting field and appears to exploit and target cells undergoing senescence—senolytics which seek to destroy senescent cells, and senomorphics which seek to suppress senescence by altering the SASP.^42^ The effectiveness of senotherapeutics, particularly senomorphics, will highlight the importance of interrogating the implications of the protumourigenic effects of the SASP and the escape from senescence.

Furthermore, while it can be demonstrated that senescence arrests proliferation, functioning as a barrier to tumourigenesis, it appears that the reliable detection methods of senescent cells *in vivo* remains challenging.^43^ It has been observed that while the various OIS biomarkers are detectable to varying degrees in preneoplastic cells as well as in the microenvironment, seemingly suggesting a complex interplay of the SASP, the sensitivities and specificity of any one biomarker in the panel were not adequate^43^ Interestingly, however, it appears that SA‐β‐Galactosidase emerged as the only biomarker reliably detectable in pancreatic preneoplastic lesions relative to pancreatic adenocarcinoma in this study.^43^ This further highlights the importance of interrogating the escape from senescence and how we can identify them *in vivo*. In this regard, Gorgoulis and colleagues recently proposed a multistep, multimarker panel of biomarkers in identifying senescent cells,^37^ with the inclusion of GL‐13 (SenTraGor), a novel tool with the capacity of being flexible and robust.^37^

**Figure 2:**
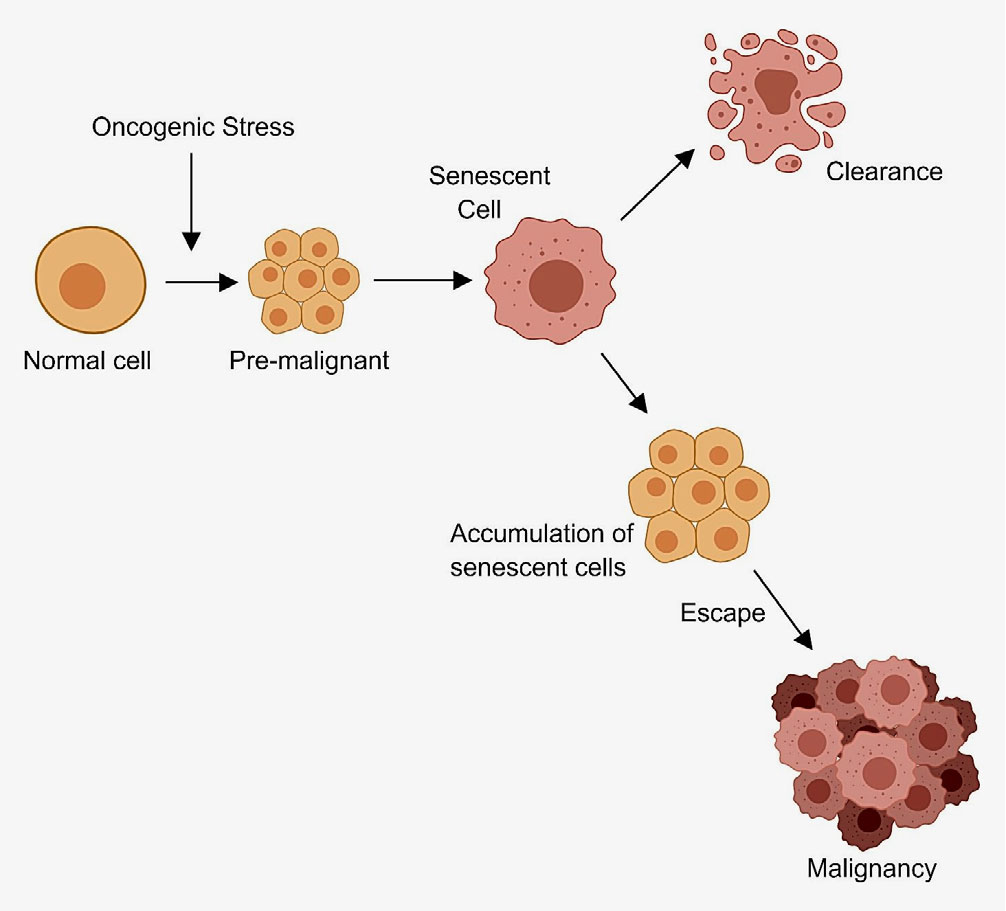
The oncogene‐induced senescence model predicts that when tumour cells undergo some form of oncogenic stress, it induces a stress‐response barrier—triggering senescence.^20^, ^22^ Progress to malignancy occurs as cells bypass or escape the antitumour barrier^14^, ^18^, ^34^—Created with BioRender.com. [Color figure can be viewed at wileyonlinelibrary.com]

So far, we can see that; senescence is characteristic of a barrier^13^, ^18^, ^20^, ^22^ (Fig. 2) and has been shown in pancreatic cancers,^35^ and Cdc6 has been associated with the induction of senescence.^13^, ^14^ Putting all this together, it will therefore be interesting to note the direct implications of Cdc6 on the induction of senescence, the mechanisms facilitating the eventual escape of cells from the senesced state leading to cancer progression, and are there, if any, exploitable pathways to senescence, knowing the Cdc6 status.

## Alternative Predictions of Mechanisms of Induction of Senescence: Subcellular Localisation of Cdc6

Cdc6 has been shown to induce senescence in ways understood to be due to its nuclear effects in inducing replicative stress and subsequently oncogene‐induced senescence.^13^, ^14^ To add to its senescence potential, there have been recent reports of cytoplasmic Cdc6 in interaction with Cyclin E inducing senescence in CSN5‐knockdown cells, as detected by SA‐β‐galactosidase (senescence marker).^44^ This is particularly exciting because to date, much has been discussed about the nuclear effects of Cdc6 and the mechanism for OIS as part of the DDR,^13^, ^14^ however, this appears to suggest an alternative potential functionality in the subcellular localisation of Cdc6. Indeed, as the cells used in our study were p53 and INK4 deficient,^44^ the findings of Ueda and colleagues seemingly suggest that the pathway taken to senescence with regards to cytoplasmic Cdc6 is a novel one as these two factors are traditionally implicated as markers of senescence.^36^

Additionally, CSN5 depletion has been observed to induce an upregulation of Cdk2, and subsequently Cyclin E in the cytoplasm, where the implication of this was the induction of senescence in a p53 deficient manner.^45^ Furthermore, it was noted that in CSN5‐deplete cells, the knockdown of Cdk2 resulted in a return of Cyclin E to normal levels and the ablation of senescence.^45^ Interestingly however, on its own, cytoplasmic Cdk2 did not appear to have a significant impact on senescence.^45^ Instead, Cyclin E exhibited a strong detection signal in cells undergoing senescence, and that cytosolically expressed Cyclin E appeared to induce senescence.^45^ Importantly, nuclear expression of Cyclin E did not induce a similar effect.^45^

Altogether these findings appear to demonstrate that the presence of Cdc6 within the cytoplasm was detected in cells with CSN5 knockout undergoing senescence, and this was associated with detection of Cyclin E in the cytoplasm as well—seemingly reinforcing the potential of an interaction between the two.^44^

**Figure 3:**
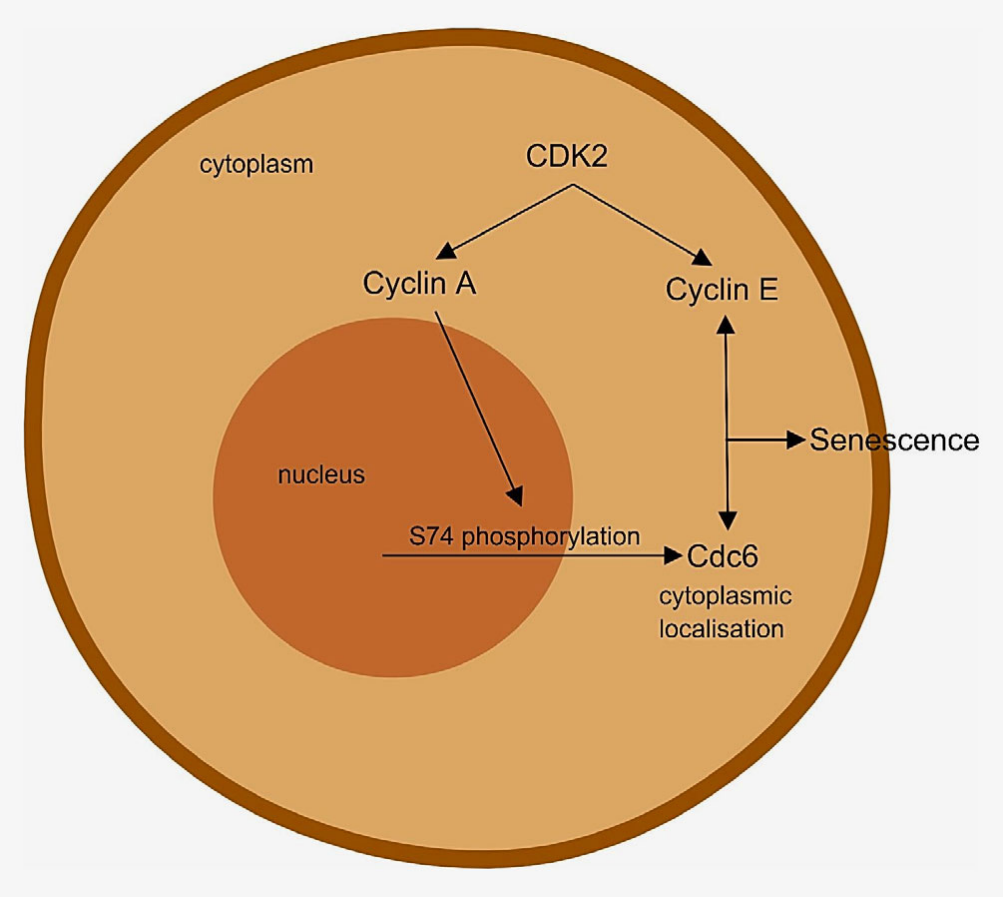
Hypothetical model for a potentially exploitable mechanism in driving the cytosolic retention of Cdc6 in inducing senescence *via* a novel manner—Created with BioRender.com. [Color figure can be viewed at wileyonlinelibrary.com]

In addition, it has also been reported that the subcellular localisation of Cdc6 can be dependent on site of phosphorylation and is largely driven by the phosphorylation of S74 mediated by Cyclin A‐Cdk2.^46^, ^47^ In context, this could potentially be an exploitable mechanism in terms of driving cytosolic retention of Cdc6 to induce senescence (Fig. 3). Yim and colleagues propose that Cdc6 may be sequentially phosphorylated at S54 by Cyclin E more effectively and remains nuclear while Cyclin A/Cdk2 phosphorylates free Cdc6 at S74 in the S phase, possibly tagging it for degradation and translocation to the cytoplasm.^47^ Taken together, this hypothesis could possibly account for the larger Cdc6 overexpression‐senescence narrative,^14^ whereby while S54 is sequentially phosphorylated and localised within the nucleus, as part of replication,^47^ there could potentially be an excess of soluble Cdc6 that becomes phosphorylated at S74 affecting cytoplasmic localisation/retention^47^—offering a specific target which affects cells overexpressing Cdc6.

It will, therefore, be interesting to extrapolate this finding by interrogating the features of CSN5 depletion, specifically that of Cdk2—Cdk2 has been shown to be related to a concomitant rise in cytoplasmic Cyclin E^45^—could there also be a similar upregulation of Cyclin A? Following on from that, could the upregulation of Cyclin A selectively drive the retention of Cdc6 in the cytoplasm? If validated, this could offer a novel opportunity to induce senescence as part of the currently unravelling novel Cdc6 cytoplasmic retention pathway. Nevertheless, there is also the likelihood that this translocation mechanism represents nothing more than merely processes within normal physiology of Cdc6 degradation, unrelated to senescence. However, as these studies indicate a plausible novel pathway to senescence driven by cytoplasmic Cdc6 and Cyclin E,^44^, ^45^ the exploration of such a mechanism is a worthy venture.

It has been proposed that novel molecules to induce the retention of Cdc6 in the cytoplasm and inhibit the CSN5–Cdk2 interaction could be beneficial in inducing senescence.^45^ A demonstrable increase in cytoplasmic retention of Cyclin A fits within our speculation. This will indicate that Cdk2‐CSN5 interaction inhibition could therefore potentially predict a double whammy of upregulating the levels of what are now believed to be key drivers in this mechanism, Cdc6 and Cyclin E, in the cytoplasm with the end effect of inducing senescence—signifying a role of Cyclin A in partaking in this pathway, possibly *via* the phosphorylation of S74.

Furthermore, such a mechanism is also shown to be p53 and INK4 independent,^44^ which will be beneficial given that progression in tumours frequently involve the inactivation of these genes, thereby presenting a potentially versatile mechanism. In addition, Serrano *et al*. have demonstrated that the interruption in p16/p53 signalling alleviated cellular arrest induced by oncogenic RAS,^36^ suggesting that a model that is devoid of p53/p16 to induce senescence will be very useful. As much of what we know about senescence bypass involves the inactivation of p53 or p16, this will help us understand how an eventual escape and attenuation of the senescence response occurs.

## Conclusion

Cancer and particularly pancreatic cancer remain a deadly disease with a dismal survival rate, beckoning a race toward improved detection and therapeutic targets of which Cdc6 shows potential. As detailed studies on Cdc6 and pancreatic cancer are few and far between, here we discussed the potential involvement of Cdc6 *via* the downstream pathways of common genetic alterations in pancreatic cancer. The oncogenic potential explained *via* the DDR is reiterated^22^ and the transcriptional effect of Cdc6 also explored, specifically that of E‐cadherin and the role within EMT.^11^, ^12^ In addition, the link between Cdc6 and senescence was interrogated. We further discussed the key position of senescence, detection methods, the interrogation of senescence escape as well as how it is being exploited currently. Specifically, we highlighted the novelty of subcellular localisation of Cdc6 in the cytoplasm in inducing senescence,^42^ of which the implications are wide. We specifically elaborated the significance of the cytoplasmic retention of Cdc6 in inducing senescence in interaction with Cyclin E.^44^ As Cyclin E is upregulated in the cytoplasm with CSN5 depletion, possibly due to upregulation of Cdk2,^45^ this review hypothesises and speculates the concomitant upregulation of Cyclin A as well. If validated, this could potentially be considered for further investigation in inducing Cdc6 cytoplasmic retention. This could also indicate a potential pathway to senescence *via* Cdc6 cytoplasmic retention by inhibition of the Cdk2‐CSN5 pathway. Furthermore, the proposed mechanism for Cdk2 cytoplasmic localisation is *via* Akt kinase, part of the RAS pathway,^45^ highlighting the potential of the CSN5‐Cdk2 interaction in this regard in inducing cytoplasmic retention in PDAC—given that KRAS mutations are very common in pancreatic cancer.^3^ This novel mechanism could also potentially aid our understanding of how senescence works and gets bypassed as this explains some of the mechanisms precluding the escape from senescence and eventual relapse given the p53 and INK4 independence.^17^, ^40^ A better understanding of the behaviour(s) of Cdc6 and how we can exploit it to our benefit could innovate strategies against cancer, particularly in view of new strategies for detecting senescence and senotherapeutics as alluded to by Gorgoulis *et al*.^37^

## Conflict of interest

Prof. Paul Townsend is cofounder of Karus Therapeutics Ltd, PRECignature, Pentagon Therapeutics (being a co‐inventor of SenTraGor) and is a NED for Aptamer Group, all UK. Nicholas Lim has no conflicts to disclose.
